# Improving the Hospital Transfer Process for Acute Type A Aortic Dissections

**DOI:** 10.7759/cureus.33451

**Published:** 2023-01-06

**Authors:** Rishi Wagle, Brooke Baumgartner, Alyssa Chen, Divya Chilukuri, Neely Helton

**Affiliations:** 1 School of Public Health, University of Texas Health Science Center at Houston, Houston, USA; 2 School of Public Health, University of Texas Health Science Center at Houston, Dallas, USA; 3 Nursing, University of Texas Southwestern Medical Center, Dallas, USA

**Keywords:** quality improvement and patient safety, safety outcomes, inter-hospital transfers, lean six-sigma, process & performance improvement, quality improvement research

## Abstract

Patients with acute type A aortic dissection who arrive at hospitals that lack the facilities to treat them must be transferred to a tertiary care facility to receive treatment. The transfer process involves a checkpoint at which the transfer is accepted or denied. Delays in making this decision may lead to suboptimal health outcomes. In light of this, the goal of this project was to devise a way to reduce the time to decision of transfer requests for patients with an acute type A aortic dissection. The project followed the Define-Measure-Analyze-Improve-Control (DMAIC) approach. To better understand the process, data were obtained from the University of Texas Southwestern Medical Center regarding reasons for patient transfer cancellation and the average time until a transfer was denied or accepted. After data analysis, a fishbone diagram was used to display 23 root causes of the delays in time to decision of the transfer request. These were narrowed down to the following four significant causes using a nominal voting technique: (1) no standard on disease-specific information for the handoff, (2) lack of a real-time database, (3) incompatible electronic health record system between facilities, and (4) multiple communication handoffs causing confusion. Solutions to each root cause were evaluated using a solution selection matrix. The final two solutions proposed for implementation were as follows: (1) to establish checklists of required documents and patient transfer criteria and (2) to create a regional database to provide real-time information on hospital capacity.

## Introduction

This project focuses on increasing the efficiency of the hospital transfer process for patients with an acute type A aortic dissection, which is an emergent heart condition. When such a patient arrives at a regional hospital or care facility that lacks the facilities to treat the condition, the patient must be transferred to a tertiary care facility. Regarding this process, there is great scope for improvement in terms of the amount of time until the transfer has either been accepted or denied. Delays in the decision can lead to negative consequences for the health of the patients needing the surgeries. Due to these delays, patients may not receive the care they need in a prompt manner, or they may waste valuable time waiting for a transfer decision that will ultimately be denied. This time could instead be used to contact another tertiary care facility. By increasing the efficiency of the transfer process in these cardiac emergencies, the process of admission leading to procedural treatment can be handled in a more efficient and timely manner.

The organization where the intervention took place is the William P. Clements Jr. University Hospital Division of Cardiology at the University of Texas (UT) Southwestern Medical Center. The Cardiology Division at UT Southwestern is ranked as the best hospital in the state of Texas for cardiology and heart surgery and as #11 in the United States [1]. The center treats approximately 600 patients every month and in order to deliver this care, UT Southwestern cardiologists focus on a team-based approach that promotes quality care, innovation, and high-level research [1]. UT Southwestern has made groundbreaking contributions to the field of cardiology and is participating in research that has the potential to transform cardiovascular health and treatment of emergent cardiac patients, contributing to facilitating even better care for patients worldwide [1].

## Technical report

“Define” phase

A project charter is a document produced at the beginning of project development in the “Define” phase of the Define-Measure-Analyze-Improve-Control (DMAIC) system for process improvement. The charter is valuable in this phase of development because it functions as an outline for the project as a whole. Thus, the creation of the charter by multiple team members allows for collaboration early on in the development of the project. By encouraging team members to work together to create a concise document with categories such as the problem statement, goals/metrics, business case, and deliverables, the creation of a charter enhances creativity and brainstorming while also directing the group to write concise descriptions for each category that lay the framework for the further creation, implementation, and analysis of the project downstream. The project charter also ensures that team members can delegate tasks to each other (which would then be documented in the charter) and can assess any areas of the project for which they may not be adequately prepared. Therefore, the charter not only assists by enumerating what the group is already aware of, but it also assists the group with finding out what tasks must be executed going forward.

Project charter

Problem

Acute type A aortic dissections are life-threatening emergencies for which guidelines recommend emergent operation where time to operation is critical for preventing damage to critical organs (brain, heart, limbs, kidneys, etc.) [2]. In the process of patient transfer, communication is repeated by the referring facility to each receiving facility until the patient is accepted, and critical time may be spent waiting on hold, repeating information, and evaluating whether the facility is a good fit for the patient. This process still occurs in the event of transfer denial and can result in even more waste of time because another tertiary facility would need to be contacted for the transfer and this process would need to be restarted. This leads to an inefficient, redundant, and wasteful process that this project will focus on improving.

Rationale

An acute type A aortic dissection is a surgical emergency. Without surgical intervention, mortality rates increase by 1-2% for each hour after symptom onset with mortality of 50% at 48 hours [3]. Thus, timely transfer is critical, and efforts should be made to reduce wasting of time and improve efficiency to improve patient outcomes.

Aim Statement

Reduce time to decision of hospital transfer request for acute type A aortic dissection by 20% overall within six months, where time to decision of transfer is defined as the time between when the call was initiated by the referring facility to when the transferring facility formally accepts or declines the request.

Expectations

Type A aortic dissections are a life-threatening condition. By improving the time to a transfer decision, patients will be able to receive care sooner, reducing the risk of mortality and lifelong complications. This project will reduce overlap in communication, reduce the amount of time spent waiting on hold, and reduce the number of transfer facilities contacted until the patient is accepted for transfer. Overall, this will create a more efficient process that will result in decreased patient mortality and complications and increased job satisfaction for providers and transfer staff.

Outcome Measure(s)

The measures to be affected as a result of this project are as follows: (1) time to accept the transfer, defined as the time between when the call is initiated by the referring facility to when the memorandum of transfer (MOT) is signed, and (2) time to transfer denial, defined as the time between when the call is initiated by the referring facility to the time when either the referring or receiving facility cancels the transfer request.

Process Measures

The measures that indicate if the parts or steps in the system are performing as planned to affect the outcome measure are as follows: average call time (an effective measure of the reduction of time being on hold), repetition of information, number of total calls, and number of facilities contacted.

Balancing Measures

The measures that will inform whether or not problems are being introduced elsewhere in the system are as follows: increasing wait time for elective operations and average time spent by the referring secretary, physician, and nurse on each transfer.

Key Stakeholders

This project will require input and support from accepting facility surgeons (vascular, cardiothoracic), emergency department (ED) physicians, transfer center nurses and staff, as well as referring facility secretaries, nurses, and physicians. Different techniques would be utilized to engage each stakeholder. Vascular surgeons will be contacted via email regarding what clinical information is relevant for accepting a transfer. ED staff will be contacted via email regarding what clinical information is relevant from an ED perspective. Transfer center and referring facility staff will be surveyed using user interviews.

Barriers

Potential barriers to success include difficulty in obtaining necessary data from a representative group of referring facilities, coordinating with multiple stakeholders, and fitting into stakeholders’ schedules. Plans to overcome these barriers include relying on the experience of transfer center staff who have interacted with many of the common referring facilities, utilizing connections in medicine and quality improvement, and emailing/contacting early.

Change Ideas

To learn more about the process or system being improved, team members will conduct user interviews with people within the process as allowed by the respective parties' schedules, analyze the data alongside a data analyst, and use diagrams like the fishbone diagram to display key information. Initial tests of change (plan-do-study-act cycles) will be evaluated using a standardized form from each tertiary care center that the referring facility staff will have before initiating the call to reduce wait time and ancillary questions. In addition, technological tools will be used, including a regional dashboard that displays the capacity, services available, and phone numbers as well as one transfer package for each patient that can be sent to each receiving facility to reduce the repetition of information by the referring facility.

Milestones and Deliverables

The overall improvement process is expected to take place over six months to achieve the goal of the aim statement. The first month would involve connecting with all stakeholders, scheduling user interviews, and establishing the project charter and the Suppliers-Inputs-Process-Outcomes-Customers (SIPOC) table. The second month would involve meeting with a data analyst to understand the data available and brainstorming the analysis phase. The second month would also serve as the time for conducting user interviews, process mapping, as well as creating a fishbone diagram. In the third month, team members would work on improvement analyses, solution planning, iterative planning and development with stakeholder input, a solution selection matrix, and a control chart to determine if any changes in the intervention need to be made. The sixth month brings the deadline for achieving the goal of the aim statement.

Suppliers-Inputs-Process-Outcomes-Consumers (SIPOC) table

**Figure 1:**
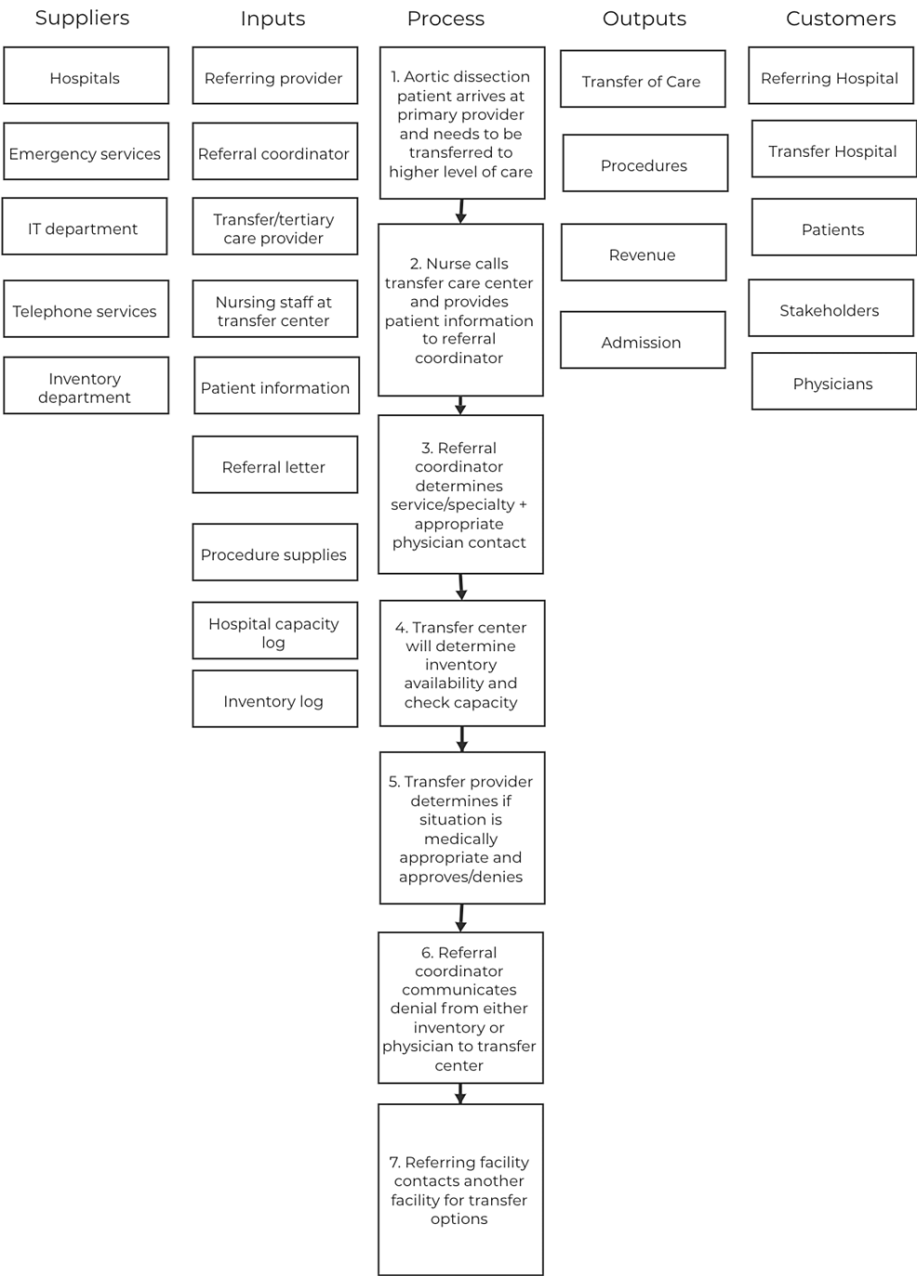
The SIPOC table created to break the process down into separate steps and simplify project variables SIPOC: Suppliers-Inputs-Process-Outputs-Consumers; IT: information technology

A SIPOC table is a process mapping tool used in process improvement, and it focuses on establishing the important aspects of the project from the beginning to the end of the process. The five components of a SIPOC table are suppliers, inputs, the process, outputs, and customers. Each component focuses on a different aspect of the process. The “Process” component of the SIPOC is often enumerated first and it details in a step-by-step progression the process that is being improved upon by the project. After the “Process”, the “Outputs” are compiled, which include everything that is produced as a result of the process including written outputs, action items that are completed, and any data that is produced as a result of the process. Following “Outputs” are the “Customers” who are the recipients of the outputs. Next comes the “Inputs”, which are everything that is necessary for the process to occur, including human resources, materials, knowledge, and anything else. Following “Inputs” are the “Suppliers”, which make up the producers of the inputs. The SIPOC is important in the Define phase because it simplifies the process into simple steps and it also simplifies the variables of the project into broad, overarching categories. This helps maintain the organization of priorities and can help elucidate the next steps moving forward. While creating the SIPOC for this project (Figure 1), the team learned that simplifying a process like the admission of an emergent cardiac patient transfer is difficult to do without involving several different personnel, each with different skills and tasks. It helped with understanding the complexity of the process and ensuring that each step in the process that is causing a bottleneck and reducing overall efficiency is tackled.

“Measure” phase

**Figure 2:**
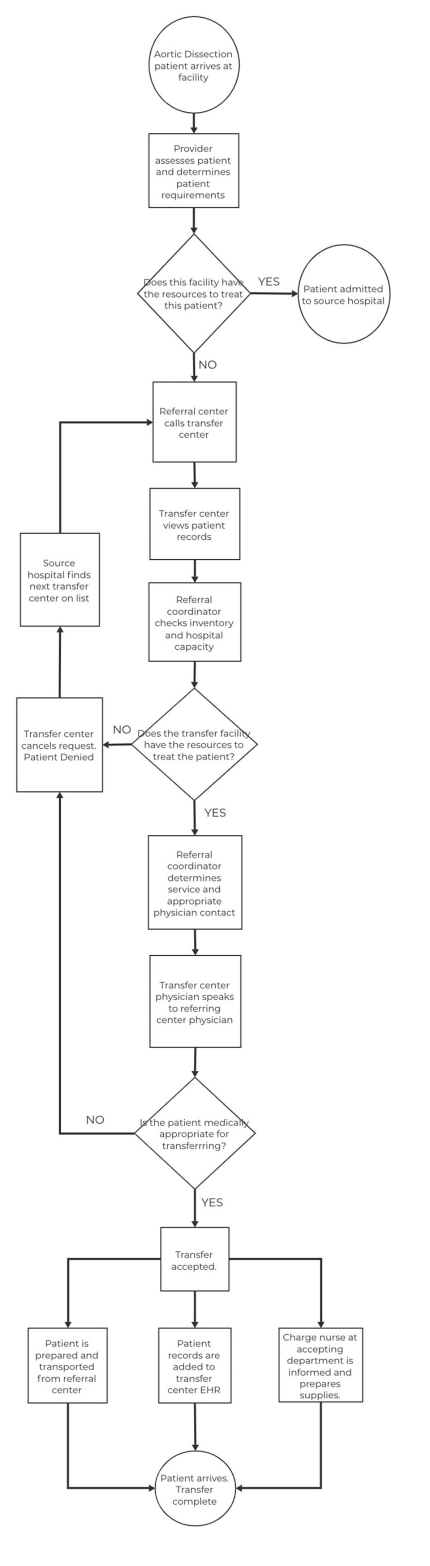
Process flow chart depicting in detail the stepwise progression of the process from beginning to end EHR: electronic health record

The process addressed by the intervention entails the transfer process between two healthcare facilities for patients with acute type A aortic dissections and other emergent heart conditions. A flow chart (Figure 2) was used to depict this transfer process. The process begins with the presentation of the aortic dissection patient to the primary healthcare provider. This provider can be present within multiple different settings including the general hospital wards/units where the patient is on the medical/surgical unit (“Meg Surg”), the medical or surgical intensive care unit (“ICU”), or within the emergency department (“ED”). Upon presentation of the aortic dissection patient to the provider in one of the settings listed above, the provider assesses the patient’s condition, makes the diagnosis of acute type A aortic dissection, and decides to transfer to a different level of care that is more appropriate given the patient’s status. The next step in the process involves the transfer phase of the process. This begins with the department nurse calling the transfer care center. Next, the transfer care center accepts the call and receives patient information from the nurse. A referral coordinator then determines the service and/or specialty that is most appropriate for the patient’s transfer and follows that by determining the appropriate physician contact at the transfer location. The transfer center then determines bed availability at the accepting facility and checks capacity alerts. The referral coordinator then communicates availability to the attending physician at the post-transfer level of care before connecting the referring provider to the accepting provider. The accepting provider then determines if the transfer is medically appropriate given the bed availability. If the transfer is deemed appropriate, the referral coordinator will call the referring facility for patient demographics and insurance information. After the information is approved, the coordinator will assign the bed to the patient being transferred and communicate the report information and patient needs to the accepting floor charge nurse. The referral coordinator will coordinate changes in patient status with the referral center and the transfer center will track the patient until they are admitted to the new location. At this point, the patient is transferred, and the process is complete. At any point in this process, the transfer can be denied for multiple reasons including capacity limits and surgeon availability.

After detailing the important actions that comprise the process, multiple points within the overall process structure were found to have areas where inefficiency could potentially exist. These inefficiencies were due to causes including repetition of information, errors in effective communication, and missed connections. Since transfer acceptance or denial follows the same process, there is an element of waste in both of these results that can be improved and potentially lead to better health outcomes. As a result of these inefficiencies in this process among others that exist within the overall healthcare system, cancellations in patient transfers begin to rise. As displayed in the Pareto chart below, the labeled reasons for cancellation within the data system vary. Regardless of the cause, pending cancellations occur over a broad range of waiting times. While some of the causes leading to the cancellation of the transfer are unable to be made more efficient (e.g. the death of a patient), the main learning point for this problem was that high values for the time to cancellation of the transfer represent a significant waste of resources for all aspects of the transfer system. This led to a focus on finding a solution that could provide benefits by reducing wait times during transfer acceptance and denial, making the transfer process more efficient for all entities involved.

**Figure 3:**
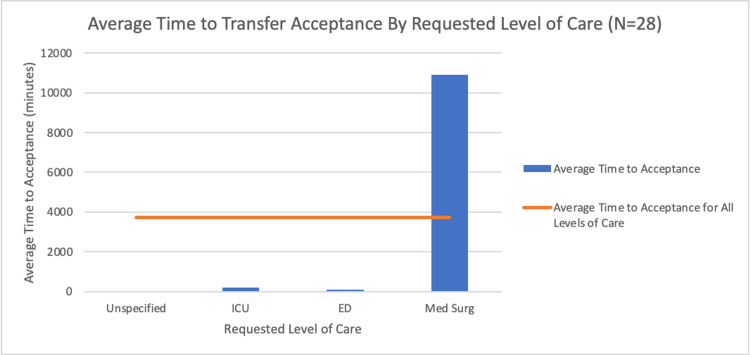
Bar chart displaying the average time to transfer acceptance grouped by the requested level of care ICU: intensive care unit; ED: emergency department; Med Surg: medical/surgical unit

**Figure 4:**
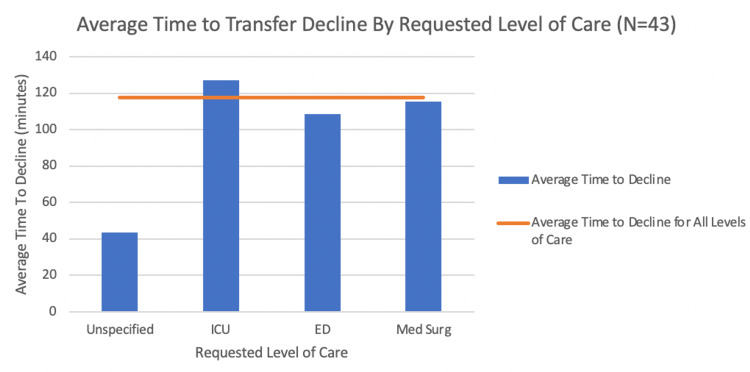
Bar chart displaying the average time to transfer decline grouped by the requested level of care ICU: intensive care unit; ED: emergency department; Med Surg: medical/surgical unit

**Figure 5:**
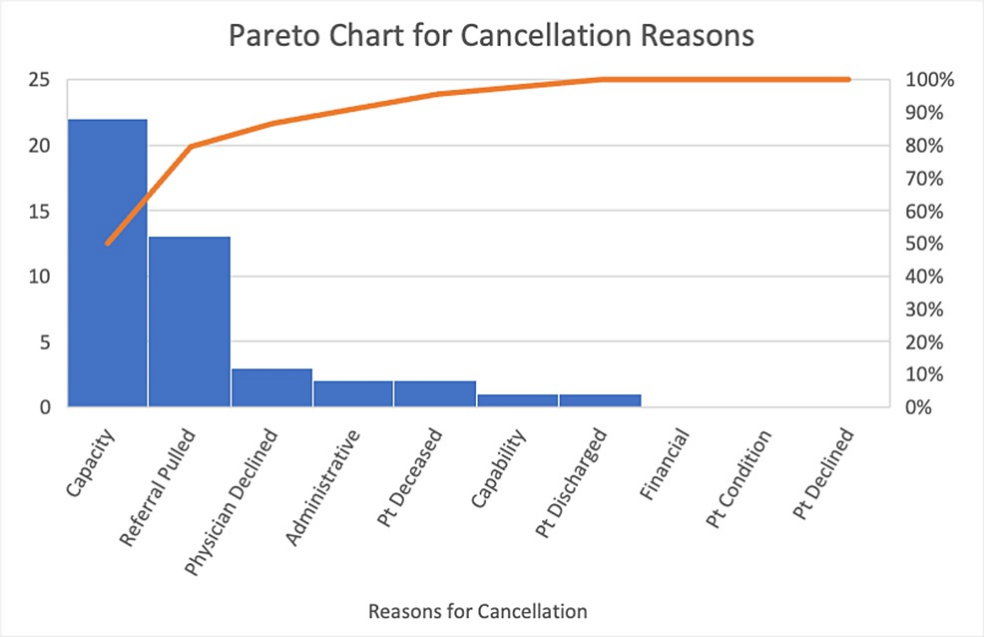
Pareto chart displaying the frequency for each reason for cancellation The orange line indicates cumulative frequencies across reasons for cancellation Pt: patient

To better understand this process, the reason for patient transfer cancellation and the average time in minutes until a transfer was denied or accepted were further examined. For accepted patients, the mean time to acceptance at the requested level of care for the ICU was approximately 212.09 minutes, the mean time for the ED was approximately 97.33 minutes, and the mean time for the Med Surg was approximately 10,897.5 minutes for a sample of 28 patients with an overall average of approximately 3,735.64 minutes until an acceptance decision was reached. These values are displayed in the bar chart in Figure 3 below. However, the extremely large value of 21,600 minutes in the “Med Surg” category skewed the overall average. When this value was removed, the Med Surg mean time was 195 minutes with an overall average of 198.70 minutes for a sample of 27 patients until a transfer was accepted. At the requested level of care, the mean time to transfer denial at the ICU was approximately 127.18 minutes, the mean time for Med Surg was approximately 115.33 minutes, the mean time for ED was approximately 108.5 minutes, and the mean time for an unspecified location was approximately 43.75 minutes for a sample of 43 patients with an overall average of 117.92 minutes until a denial decision was reached. The values are displayed in the bar chart in Figure 4 below. The primary reason for transfer patient cancellation, as seen in Figure 5 below, is capacity issues, followed by the referral being pulled, the physician declining, administrative issues, patient death, capability problems, the patient being discharged, financial difficulties, the patient conditions, and the patient choosing to decline. As reflected in these results, there is much room for improvement in the transfer process that can be achieved with the establishment of a more efficient protocol.

The selection of tools was limited by the data available, which began to be collected only in 2020. The data variables available from the UT Southwestern transfer center included temporal data on when the transfer started and when the transfer was accepted or declined, diagnosis, the reason for transfer, the service requested, level of care requested, the current level of care at the referral facility, the name of the procedure requested, and reasons for cancellation. To work towards the aim of reducing the number of cancellations, reasons for cancellations were first investigated with the Pareto chart (Figure 5) to categorize the reasons and visually determine what the most common reasons for cancellations were. Secondly, the team wished to understand if there was a difference in cancellations depending on the severity of the transfer. This was investigated with a bar chart (Figure 4) to compare the average time that it took to decline a transfer based on the level of care that was requested and the current level of care at the referring facility.

The greatest challenge with the data was that some of the categories, such as “reason for cancellation,” were text responses that were not all uniform, which necessitated the manual separation of the data. There were also fewer inputs in the selected condition than anticipated, and hence there was potential for the data to be heavily skewed by outliers leading to an inability to show an accurate representation of time to transfer decision. Due to patient confidentiality and Health Insurance Portability and Accountability Act (HIPAA) regulations, there were also many inputs that were “NULL” and had to be removed from consideration. Regarding transfer acceptance data, there were no timestamps available for the different phone calls and checks that occur within this process, and hence it was difficult to determine if there was a certain point in the process that led to excessive delays. When evaluating this process, the team learned about the difficulties that a transfer center faces, the main issues and causes associated with patient transfer cancellation, and the transfer wait times associated with the different levels of care.

“Analyze” phase

**Figure 6:**
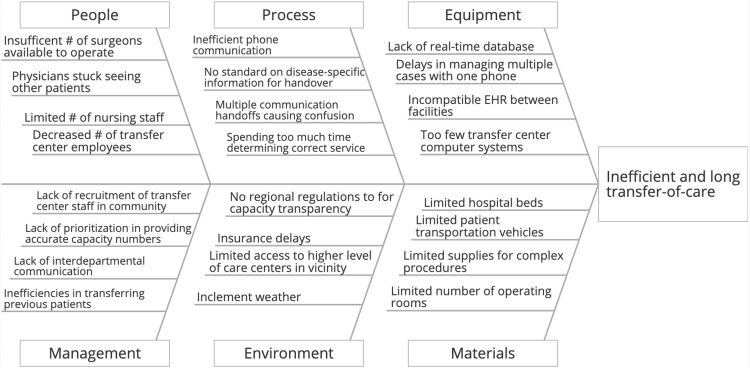
The fishbone diagram: it is a tool used to display all possible root causes of the problem (i.e., inefficient and long transfer-of-care) grouped into six separate categories EHR: electronic health record

A fishbone diagram is a type of diagram used to convey possible causes of a problem or negative outcome. It is designed with the problem at hand on the right side (or at the fish’s head) with the spine of the fish connecting each of the potential causes of the problem to the problem itself. The branches off of the spine represent categories within which each potential cause exists, which can include materials, people, processes, and others. The purpose of this diagram is to show all of the possible groups of causes that could be causing the problem and to help the problem solvers elucidate as many potential causes as possible. It is of particular use in helping to understand the causes because of the categorization of each cause into one of a limited number of categories (in this case, six); this helps the team recognize how each potential cause affects any other potential causes and where the root of the problem may lie. The data to complete the fishbone was gathered by brainstorming upon analysis of the data that was received from UT Southwestern Medical Center transfer center and the graphs that were made using the data including bar and Pareto charts. The entire quality improvement team participated in the brainstorming session to complete the fishbone for this project (Figure 6). Upon receiving input from each of the participants, the team learned about how each member interpreted the data including the various reasons for cancellation and the way in which each reason for the cancellation was due to an issue at some point within the process that was outlined in the flow diagram. The team also learned about different variables not found in the data that had not previously been considered as being part of the root cause that could lead to delays in decision-making including the differences between electronic health record systems (EHRs) between hospitals and the apparent lack of real-time transfer databases and standardization of information needing to be relayed, which are necessary for fast and efficient decision-making during a transfer of care.

The secondary tool that was used for narrowing down the root causes from the fishbone diagram was the nominal group voting technique. This tool was selected because it was the best way to incorporate each team member's opinions regarding the root causes from the fishbone diagram in a way that provided an equal voice, in the form of a vote, for each member. The nominal group technique functions by listing out all causes in the fishbone diagram in a table and having each member of the group place their votes for the most important causes; in the case of this team, there were 23 causes present with each team member being given 12 votes to cast. Upon determining the seven causes with the highest number of total votes, the technique was utilized once more, but this time with the top five most picked causes from the first iteration and with a ranking system used for voting with 1 as the least important cause and 5 as the most important cause (Table 1). Each team member ranked each of the five causes and the total points were tallied for each cause, which allowed the team to determine what were felt collectively to be the most important causes to focus on. The root causes identified through the completion of the fishbone diagram and the nominal group technique were as follows: (1) there is no standard on disease-specific information for handover between facilities, (2) there is a lack of a real-time database for referring facilities to know if tertiary care centers are accepting transfer patients, (3) incompatible EHRs make it difficult to transfer health information between these facilities, and (4) the multiple communication handoffs in this process cause confusion. Overall, a lack of regulation and standards on how this process is handled lies at the root of why there are inefficiencies in the transferring of acute type A aortic dissection patients.

**Table 1:** Table displaying the results of the second iteration of the nominal group voting technique 1: least important; 5: most important

Cause	Team member 1	Team member 2	Team member 3	Team member 4	Total
A. No standard on disease-specific information for handover	5	5	5	5	20
B. Incompatible EHR between facilities	2	3	3	4	12
C. Limited number of hospital beds	1	1	1	1	4
D. Lack of a real-time database	4	4	4	3	15
E. Multiple communication handoffs causing confusion	3	2	2	2	9

“Improve” phase

The solution selection matrix was a key part of the “Improve” phase because it allowed the team to have a methodology on hand for determining the best solution based on what was learned from the “Analyze” phase. The most important part of the solution selection matrix was the ability to quantify the value of each criterion based on a specialized understanding of the issues at hand that needed to be overcome to reach the end goal. By having numerical data that was based on an understanding of the process being improved, the solution selection matrix represented a way for the team to come to a conclusion about how the process could best be improved using a combination of objectivity and personal knowledge. This helped to eliminate potential biases and include all important differentiating criteria. The presentation of the solution selection matrix in an easily understandable format with definitive rankings on the right side of the table was also beneficial because it laid out the solutions in order from most effective to least effective (based on the criteria listed and their value in terms of accomplishing the end goal).

The process began by examining the root causes identified in the “Analyze” phase. Based on these four root causes, team members were able to identify seven solutions relating to the aim statement: (1) the creation of a database that contains disease-specific criteria for transfer, (2) a real-time intrahospital database that allows the transfer center to easily see the capacity of the hospital, (3) a real-time interhospital database that allows other facilities to see the capacity of tertiary facilities, (4) the creation of a protocol of communication order in this process, (5) the creation of a checklist of required documents needed for transfer, (6) an agreement to share protected health information (PHI) across EHRs, and (7) the creation of a checklist of patient transfer criteria needed for the transfer to be completed. These were each selected because each was deemed to have the potential to reduce the time to decision of transfer patients, they addressed the root causes, and because these protocols are not currently in place at UT Southwestern and the surrounding areas.

**Figure 7:**
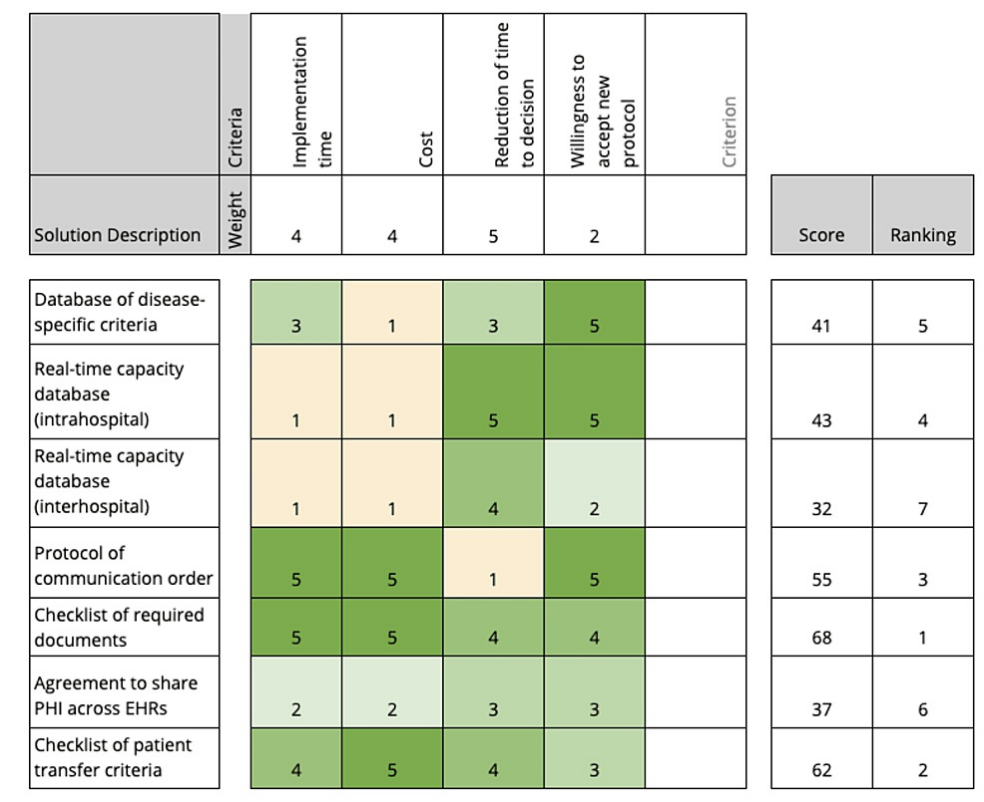
The solution selection matrix that was used to evaluate and rank each potential solution using weighted criteria The methodology for weighting of criteria can be found in Table 2 PHI: protected health information; EHR: electronic health record

Next, criteria were selected that would be used for rating these solutions. The four criteria selected for the matrix were as follows: (1) the time to implement the solution, (2) the cost of implementing the solution, (3) the impact the solution would have on reducing the time to a decision, and (4) the willingness of the referring and receiving facilities to implement the solution. Referring back to the aim statement, the reduction of time to a decision was identified as the most important criterion and hence this was weighted the heaviest at 5, followed by implementation time and cost, which were both weighted at 4, and lastly, willingness to implement new protocol was weighted at 2. The team determined that the best implementations to address the overarching problem will be able to be established quickly, at a relatively low cost, will have large support from both the referring and receiving facility staff, and will have a large impact in reducing the time to a decision. Based on these goals, weight descriptions for each criterion were made, which can be viewed in Table 2 below. Through team collaboration, each solution was evaluated and placed according to the weight descriptions for the criteria. Based on the results seen in Figure 7, the top-rated solution was the checklist of all required documents necessary for transfer, followed by the checklist of necessary patient criteria, a protocol of communication order, an intrahospital real-time capacity database, a database with disease-specific criteria, an agreement to share PHI across EHRs, and lastly an interhospital real-time capacity database.

**Table 2:** Table displaying the methodology for weighting of criteria for the solution selection matrix The solution selection matrix can be found in Figure 7

Weight descriptions	1: Lowest	2	3	4	5: Highest
Implementation time	Greater than 12 months	8-12 months	4-8 months	1-4 months	Less than 1 month
Cost	Greater than $500,000	$350,000 to $500,000	$250,000 to $350,000	$100,000 to $250,000	Less than $100,000
Reduction of time to decision	Less than 5% time reduction	Reduction by 5% to 10%	Reduction by 10% to 15%	Reduction by 15% to 20%	Reduction by more than 20%
Willingness to accept intervention	Less than 30%	30% to 50%	50% to 70%	70% to 90%	Greater than 90%

As previously mentioned, the most recommended improvement strategies to increase the efficiency of transfer-of-care disposition are the use of checklists of transfer documents and patient criteria. The effects of these improvements must be analyzed in both short-term and long-term settings. In the short term, the checklists can be rolled out in relatively little time with a very little cost associated with them; however, the willingness to implement is a potential issue that was identified. Since employees in large healthcare systems are accustomed to a specific workflow, the redundancy of the checklist can be inconvenient to add to a previously established routine. However, in the long run, once the staff becomes accustomed to this addition and is able to follow it thoroughly, there will be a drastic change in reducing the decision time. Standardizing this part of the process reduces error and prevents inefficiencies in obtaining transfer-related patient referral information. Similarly, the next ranked recommendation to create a calling order protocol would lead to the same short-term effects in that it will take time for staff to become accustomed to a new system. However, it is ranked lower because it does not have a large enough benefit in reducing time to a decision because that is a knowledge-based process that requires access to information. The next recommendation is to create a database with disease-specific criteria and an agreement to share PHI across EHRs. In the short term, the database and PHI access improve the knowledge base of the staff involved and facilitate improved decision-making. However, they take a considerable amount of time to implement due to the need to research all the information for the database and the requirement of adhering to bureaucratic rules for the sharing of PHI across systems. The cost is also a huge factor because these recommendations involve more manpower including researchers and technical consultants for data sharing. Fortunately, they are moderately beneficial in addressing the ultimate goal of reducing transfer time in the long run. Lastly, the real-time capacity databases, whether intrahospital or interhospital, are the most beneficial in reducing time to a decision because they potentially allow transfer center employees to have access to information in real time without delay. However, setting up a system like this involves spending more resources in time, money, and manpower. Overall, the checklists have the most beneficial short-term and long-term effects and therefore represent the team's most recommended improvement.

Implementation of these protocols would hold great value for both the patient and the organization. As mentioned in the project charter, the risk of complication in acute type A aortic dissection increases every hour and time is critical when dealing with this condition [2]. Reducing time to a decision will lead to more efficient transfers between facilities, better patient outcomes, and allow the referring facility to contact another receiving facility more quickly if the transfer ends up being declined, leading to a reduction of mortality in these patients. These solutions will also help to reduce the amount of time spent by transfer center nurses trying to facilitate these transfers, reduce the number of phone calls and time on hold during this process, reduce the number of facilities that need to be contacted to see if they have the capacity to accept patients, as well as reduce waste in the collection and transfer of patient data. Time is critical for healthcare organizations and patients, and hence the implementation of these solutions would be beneficial for all parties and stakeholders involved in this process.

Validation of these solutions is an important step to ensure that their implementation will address the root causes identified. The team collaboratively brainstormed solutions with the root causes in mind. For example, following nominal group technique voting, the main root cause identified was the lack of a standard on disease-specific information for handovers. A checklist was proposed to standardize disease-specific patient information as well as a database to display such criteria for each disease prior to transfer. These solutions will address the lack of a standard and reduce the redundancy in communication that increases wait time to ultimately reduce the time to decision of transfer. Due to time constraints and scheduling limitations, the team served as a proxy for the stakeholders involved in the transfer process after meeting with various stakeholders to obtain an understanding of their pain points and role in the process. Prior to implementing these solutions, it is important to receive stakeholder approval to affirm that introducing such solutions into their existing process will address the various root causes.

“Control” phase

The control phase represents the final phase of the DMAIC process improvement framework. The goal of the control phase is to control the process once the improvement has been executed in order to maintain the improvements in achieving the endpoints of the process. This phase can take several weeks to be completed as it involves creating control charts to monitor how the process is being improved over time and formulating a plan to manage any potential future failures in the process as it continues to be implemented over time. Another important aspect of the control phase is the creation of a plan that emphasizes the continued use of process improvement tools to further improve the process beyond the timeframe of the current initiative. While the emphasis in a process improvement project is mostly focused on the current solution, the process will continue to be implemented by the organization for years to come. As a result, as the organization changes over time, future employees and process improvers should be provided with a plan that assists them in streamlining the process in a continuous improvement cycle over time. This plan should be created and/or updated within the control phase and represents a significant part of the process improvement cycle by emphasizing that process improvement is less of a one-time initiative and more of a culture shift that must be enriched and maintained in the long run.

The control phase is a critical step to ensure that improvements in the process are sustained and behaving as intended. For the top-ranked proposed solutions - (1) a checklist of required documents needed for transfer and (2) a checklist of patient transfer criteria needed for the transfer to be completed - the control plan is detailed in Table 3 below, which includes measuring the percentage of checklists completed for the number of transfers completed and graphically plotting this percentage via a p-chart. If the percentage sharply decreases or increases above 100%, then this serves as a quantitative indication that the checklists are underutilized or overperformed, respectively. In addition, to ensure that the checklists are behaving appropriately, measurement of the proportion of checklists that were incomplete out of all the checklists that were turned in was proposed. As a proxy of the efficiency of the overall process, the average time to decision of transfer will continue to be measured. Any variation that occurs outside the limits of reasonable statistical variation should be further evaluated to ensure all steps in the process are occurring as intended.

**Table 3:** The control plan representing the plan for continued evaluation and maintenance of the improved process beyond the time constraints of the initial quality improvement initiative EHR: electronic health record; UT Southwestern: University of Texas Southwestern Medical Center

Metric	Process owner	Monitoring	Goal	Response plan	Next level of support
Percentage of checklists completed compared to the number of transfers that were accepted	Referring center team manager	EHR	The checklist will be completed in 90% of accepted transfers	If the percentage of checklists completed falls below 50%, the team manager will remind staff of duties and provide instructions on where to find checklist information	UT Southwestern transfer center team manager will publish acceptance statistics so that referring center staff are incentivized to complete checklists
Percentage of checklists that were not complete but submitted	Referring center team manager	EHR	Less than 10% of accepted checklists will be incomplete	If the percentage of incomplete checklists is greater than 50%, the team manager will provide staff training on how to fully complete the checklist and identify barriers	UT Southwestern transfer center team manager will update the requirement criteria
Average time to decision of transfer	Transfer center data analyst	Transfer center time log	The average time to transfer decision will be reduced by 20% within twelve months after the solutions have been implemented	If the time of decision is not reduced by at least 5% in the first six months, the transfer center data analyst will do a longitudinal analysis of the reasons for the delay and identify strategies that are more time-efficient	Transfer center team manager

## Discussion

Acute type A aortic dissections are medical emergencies that require timely surgical intervention for optimal health outcomes [2]. The transfer of such patients to tertiary care centers can be necessary when these patients arrive at local community hospitals without the capabilities to adequately treat the condition. In addition, the act of interhospital transfer of acute type A aortic dissection patients has not been found to influence the surgical outcomes of the patients [4]. Therefore, such patients may be transferred to tertiary care centers from other hospitals also capable of treating this condition in order to place them under the care of veteran cardiac surgeons and surgery teams that are available at these tertiary facilities [4]. Tertiary care centers (e.g., UT Southwestern Medical Center) receive high volumes of patients [1], and patients who receive treatment for acute type A aortic dissections at higher volume hospitals have been shown to have a significantly lower operative mortality rate than those who receive treatment at low-volume hospitals [5]. Regardless of the rationale behind the transfer, increasing the efficiency of the transfer process represents an opportunity for patients to receive necessary care in a timely and prompt manner. A similar initiative involving the transfer of ST-elevation myocardial infarction (STEMI) patients was found to improve process metrics and mortality rates for patients who were transferred from outside hospitals to a tertiary care facility [6].

Against this backdrop, this quality improvement project aimed to identify lags in the time it takes to transfer acute type A aortic dissection patients and develop possible solutions to reduce this time. To that end, the team explored inefficiencies in the current transfer process at a large tertiary care center, identified root causes for these delays, and brainstormed possible solutions, such as creating checklists with required patient information and documents or a regional database with live updates of hospital capacity. While the focus of this project was improving the transfer process between facilities, further research could be conducted to determine potential solutions for increasing the efficiency of transfer of acute type A aortic dissection patients within a hospital between units (i.e., intrahospital transfer). In particular, a checklist with the required information for the transfer represents a low-cost and timely solution that has the potential to make an immediate positive impact on transfer efficiency within or between hospitals. This is supported by a study that found that the use of a checklist for the intrahospital transfer of stroke patients between the neurocritical care unit and the hospital ward led to a decreased median length of stay in the hospital and decreased time for transferring patients [7]. The biggest limitation encountered pertained to the availability of the relevant data. Due to patient confidentiality laws, much of the received data was redacted. In addition, the sample size was small as the transfer center had only collected two years of data to date, which narrowed the scope for generalizability. The limited granularity of the data also restricted the level of analysis of the processes. The strengths of this project included collaboration with the transfer center coordinator and data analysts at the UT Southwestern transfer center to discuss the current process and identify pain points as well as working efficiently together as a team. If this project were to be performed again, it would be beneficial to include data from more hospitals across Texas to create solutions that would be more widely applicable as well as increase the amount of data available. Throughout this process, the team learned about the value of quality improvement in organizations and the thought process that goes into the justification, development, and implementation of a large improvement concept such as the one that was developed.

## Conclusions

We believe that his quality improvement project focusing on improving the process of transferring patients with an acute type A aortic dissection to receive life-saving treatment will have a strongly positive impact on patient outcomes and hospital transfer efficiency if implemented. The use of Lean Six Sigma methodologies such as DMAIC was instrumental in driving the project towards effecting real change in the transfer process. Lean Six Sigma tools for quality and process improvement should be utilized in improving as many processes within and between healthcare systems as possible. The control phase in particular represents opportunities for the process to be maintained and further improved upon in the future, beyond the limited timeframe of the current project. As the American healthcare system continues to become more complex, quality improvement initiatives by healthcare administrators are needed to maintain the efficiency of processes, especially those that directly impact the health outcomes of patients suffering from emergent conditions like aortic dissections. More research would be beneficial for evaluating how standardization and technology can improve the efficiency of care for other serious medical conditions.
